# The effect of aspirin and eicosapentaenoic acid on urinary biomarkers of prostaglandin E_2_ synthesis and platelet activation in participants of the seAFOod polyp prevention trial

**DOI:** 10.1002/ijc.34764

**Published:** 2023-10-19

**Authors:** Ge Sun, Harriett Fuller, Hayley Fenton, Amanda D. Race, Amy Downing, Elizabeth A. Williams, Colin J. Rees, Louise C. Brown, Paul M. Loadman, Mark A. Hull

**Affiliations:** ^1^ Leeds Institute of Medical Research University of Leeds Leeds UK; ^2^ Institute of Cancer Therapeutics University of Bradford Bradford UK; ^3^ School of Medicine and Population Health University of Sheffield Sheffield UK; ^4^ Population Health Sciences Institute Newcastle University Newcastle upon Tyne UK; ^5^ MRC Clinical Trials Unit at University College London UK

**Keywords:** aspirin, colorectal cancer, eicosapentaenoic acid, prostaglandin, thromboxane

## Abstract

Urinary prostaglandin (PG) E metabolite (PGE‐M) and 11‐dehydro (d)‐thromboxane (TX) B_2_ are biomarkers of cyclooxygenase‐dependent prostanoid synthesis. We investigated (1) the effect of aspirin 300 mg daily and eicosapentaenoic acid (EPA) 2000 mg daily, alone and in combination, on urinary biomarker levels and, (2) whether urinary biomarker levels predicted colorectal polyp risk, during participation in the seAFOod polyp prevention trial. Urinary PGE‐M and 11‐d‐TXB_2_ were measured by liquid chromatography‐tandem mass spectrometry. The relationship between urinary biomarker levels and colorectal polyp outcomes was investigated using negative binomial (polyp number) and logistic (% with one or more polyps) regression models. Despite wide temporal variability in PGE‐M and 11‐d‐TXB_2_ levels within individuals, both aspirin and, to a lesser extent, EPA decreased levels of both biomarkers (74% [*P* ≤ .001] and 8% [*P* ≤ .05] reduction in median 11‐d‐TXB_2_ values, respectively). In the placebo group, a high (quartile [Q] 2‐4) baseline 11‐d‐TXB_2_ level predicted increased polyp number (incidence rate ratio [IRR] [95% CI] 2.26 [1.11,4.58]) and risk (odds ratio [95% CI] 3.56 [1.09,11.63]). A low (Q1) on‐treatment 11‐d‐TXB_2_ level predicted reduced colorectal polyp number compared to placebo (IRR 0.34 [0.12,0.93] for combination aspirin and EPA treatment) compared to high on‐treatment 11‐d‐TXB_2_ values (0.61 [0.34,1.11]). Aspirin and EPA both inhibit PGE‐M and 11‐d‐TXB_2_ synthesis in keeping with shared in vivo cyclooxygenase inhibition. Colorectal polyp risk and treatment response prediction by 11‐d‐TXB_2_ is consistent with a role for platelet activation during early colorectal carcinogenesis. The use of urinary 11‐d‐TXB_2_ measurement for a precision approach to colorectal cancer risk prediction and chemoprevention requires prospective evaluation.

## Graphical abstract

What's new?

In this secondary analysis of the seAFOod polyp prevention trial, the authors report that the putative colorectal cancer chemoprevention agents, aspirin and eicosapentaenoic acid reduce the levels of urinary biomarkers PGE‐M and 11‐dehydro‐TXB_2_, a finding compatible with in vivo cyclooxygenase inhibition. Moreover, urinary 11‐dehydro‐TXB_2_ levels predict both the risk of developing colorectal polyps and the chemoprevention efficacy of aspirin and eicosapentaenoic acid, consistent with a role for platelet activation during early colorectal carcinogenesis. The prospective evaluation of urinary 11‐dehydro‐TXB_2_ as a risk and therapeutic response biomarker may help in devising a precision approach to colorectal cancer prevention.

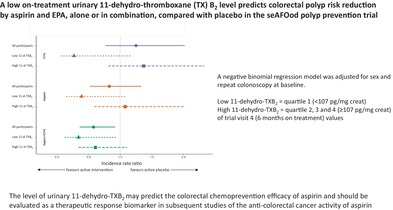

**Abbreviations**
AFPPS: Aspirin/Folate Polyp Prevention Study
ANOVA: analysis of variance
BCSP: Bowel Cancer Screening Programme
COX: cyclooxygenase
Cr: creatinine
CRC: colorectal cancer
d: dehydro
DHA: docosahexaenoic acid
EPA: eicosapentaenoic acid
HETE: hydroxyeicosatetraenoate
IMP: Investigational Medicinal Product
IQR: interquartile range
IRR: incidence rate ratio
LC‐MS/MS: liquid chromatography‐tandem mass spectrometry
LOD: limit of detection
MP: mobile phase
MRM: multiple reaction monitoring
OR: odds ratio
PDR: polyp detection rate
PG: prostaglandin
PGE‐M: prostaglandin E metabolite
PUFA: polyunsaturated fatty acid
Q: quartile
RBC: red blood cell
ROC: receiver‐operating characteristic curve
TCPS: Tennessee Colorectal Polyp Study
TX: thromboxane
u: urinary
UPLC: ultraperformance liquid chromatography
V: visit

## 1 INTRODUCTION

The seAFOod polyp prevention trial was a randomised, double‐blind, placebo‐controlled, 2 × 2 factorial trial of the colorectal cancer (CRC) chemoprevention efficacy of aspirin 300 mg daily and/or the omega‐3 polyunsaturated fatty acid (PUFA) *n*‐3C20:5 eicosapentaenoic acid (EPA) 2000 mg daily, for 12 months in ‘high risk’ (≥5 polyps <10 mm in size, or ≥3 polyps if one ≥10 mm in size) patients undergoing colonoscopy surveillance in the English Bowel Cancer Screening Programme (BCSP).^1^, ^2^

Although aspirin and EPA did not reduce colorectal polyp incidence, measured as the ‘adenoma detection rate’ (the % number of individuals with one or more colorectal polyps 12 months after clearance colonoscopy),^1^, ^2^ aspirin use was associated with a significant 22% reduction in overall colorectal polyp risk (measured as mean polyp number per participant),^1^, ^2^ consistent with a large body of evidence supporting the CRC chemoprevention activity of aspirin.^3^ There was also colorectal site‐ and polyp type (adenomatous vs serrated polyp)‐specific chemoprevention activity of aspirin and EPA; randomisation to aspirin was associated with reduced risk of serrated lesions, unlike EPA treatment, which was associated with a statistically significant reduction in risk of left‐sided (distal to the splenic flexure) adenomatous polyps only.^1^, ^2^ Subsequent ‘inside the table’ analysis of the four treatment groups within the 2 × 2 factorial trial design demonstrated that colorectal polyp risk was significantly lower in participants randomised to combination aspirin and EPA compared to either agent alone.^4^

Aspirin and EPA both inhibit the two cyclooxygenase (COX) isoforms (COX‐1 and COX‐2), which control the rate‐limiting step in the biosynthesis of several prostanoids including prostaglandin (PG) E_2_, a lipid mediator with paracrine protumorigenic and immunosuppressive activity, and thromboxane (TX) A_2_ that drives platelet aggregation.^5^, ^6^

Randomised clinical trials have demonstrated that aspirin and EPA reduce levels of the stable urinary metabolite of PGE_2_, 11α‐hydroxy‐9,15‐dioxo‐2,3,4,5‐tetranor‐prostane‐1,20‐dioic acid (commonly known as PGE‐M) in humans with a prior history of colorectal polyps.^7^, ^8^, ^9^ High urinary (u) PGE‐M levels predicted the detection of ‘advanced’ colorectal adenomatous polyps and the overall CRC chemoprevention benefit of aspirin in the Nurses' Health Study.^10^

The stable urinary metabolite of TXA_2_, 11‐dehydro (d)‐TXB_2_ is an established measure of platelet activation^11^ and of COX‐1‐dependent antiplatelet activity of aspirin,^12^ as well as, to a lesser extent, of EPA.^13^ Aspirin (at 81 mg and 325 mg daily doses) has been demonstrated to reduce 11‐d‐TXB_2_ levels in a secondary analysis of the Aspirin/Folate Polyp Prevention Study (AFPPS).^9^ Colorectal cancer patients display enhanced platelet activation measured by u11‐d‐TXB_2_ levels.^14^ However, the use of u11‐d‐TXB_2_ levels for prediction of the anti‐CRC activity of aspirin has not been investigated.

Our study aimed to characterise the effect of aspirin and EPA, alone and in combination, on urinary biomarkers of prostanoid synthesis (uPGE‐M and u11‐d‐TXB_2_), as well as investigate the role of these urinary biomarkers as predictive biomarkers of colorectal polyp risk and chemoprevention efficacy of aspirin and EPA in ‘high risk’ individuals who participated in the seAFOod polyp prevention trial.

## 2 METHODS

### 2.1 The seAFOod polyp prevention trial and biobank

The design and colorectal polyp outcomes of the seAFOod polyp prevention trial have been published in detail.^1^, ^2^ All participants in the seAFOod trial were individuals with ‘high risk’ colorectal polyp findings (≥5 polyps <10 mm in size, or ≥ 3 polyps if one ≥10 mm in size), but no synchronous CRC, at clearance screening colonoscopy.^1^, ^2^ This patient population is known to be at elevated risk of metachronous CRC compared to an age‐ and sex‐matched general population.^15^

Seven hundred and seven individuals were randomly allocated to treatment with either placebos only, aspirin 300 mg daily, EPA 2000 mg daily, or both active interventions, for 12 months, according to a double‐blind, 2 × 2 factorial design.^1^, ^2^

For this secondary trial analysis, adenomatous and serrated polyps were combined as total colorectal polyps, with ‘advanced’ colorectal polyp defined as ≥10 mm in size and/or with high‐grade dysplasia, consistent with current UK colonoscopy surveillance guidelines that acknowledge the malignant potential of serrated lesions, as well as adenomatous polyps.^15^

A urine sample was obtained at randomisation prior to starting the trial intervention (visit 1 [V1]), which was at least 7 days after BCSP screening colonoscopy; at 6 months (midtreatment during a scheduled trial visit to obtain more Investigational Medicinal Product [IMP] (visit 4 [V4]); and the day after the final treatment dose, which was the same day as the trial exit colonoscopy (visit 6 [V6]), approximately 12 months after screening colonoscopy.^2^, ^16^ Samples were immediately stored at −20°C locally, prior to transfer on dry ice to the trial Biobank for long‐term storage at −80°C.^2^ Sample collection compliance was excellent leading to curation of 78% of expected urine samples in the seAFOod trial biobank.^2^

### 2.2 Laboratory methods

Liquid chromatography‐tandem mass spectrometry (LC‐MS/MS) was performed on an Acquity H‐Class Ultra‐Performance LC (UPLC) System linked with a Xevo TQ‐XS (Waters Corp., Milford, USA) tandem quadrupole mass spectrometer operated in multiple reaction monitoring (MRM) mode.

PGE‐M and 11‐d‐TXB_2_ were extracted simultaneously from urine samples using a solid phase extraction technique. PGE‐M‐d6 and 11‐d‐TXB_2_‐d4 (both 40 μL of a 1 μg/mL solution [Cayman Chemical, Ann Arbor, MI]) were added to 1 mL of urine, which was acidified with 100 μL 1% acetic acid. Solid phase extraction cartridges (Bond Elut C18, 100 mg, 1 mL, Agilent Technologies, Stockport, UK) were preconditioned with methanol followed by acidified water (pH 3, acetic acid). The sample was applied to the column and then washed with acidified water followed by heptane. The samples were eluted in 1 mL ethyl acetate and evaporated to dryness using a Genevac EZ‐2 evaporation system. Samples were reconstituted in 50 μL 50% mobile phase (MP)A:50% MPB (composition of each MP described below), with 2 μL injected into the UPLC‐MS/MS system.

UPLC separation was achieved using an Acquity UPLC BEH C18 Column, 2.1 × 150 mm, 1.7 mm (Waters Corp.), MPA (0.1% [v/v] acetic acid [95 parts]:acetonitrile [5 parts]) and MPB (0.1% [v/v] acetic acid [10 parts]:acetonitrile [90 parts]). Each sample was analysed twice; for PGE‐M and for 11‐d‐TXB_2_. An aliquot for PGE‐M measurement was separated by a gradient of 98%‐91.5% MPA over 10 min at 500 μL/min, while the aliquot for 11‐d‐TXB_2_ measurement was separated with a gradient of 85%‐48.3% MPA over 10 minutes at 500 μL/min. Each injection was 20 minutes to allow for column re‐equilibration to the appropriate start conditions. The Xevo mass spectrometer was operated in negative electrospray ionisation mode with a capillary voltage of 2.5 kV, source temperature of 150°C, and desolvation gas flow of 1000 L/h. Cone voltage and collision energy for all analytes were established as 10 V and 15 V respectively. MRM channels were set as PGE‐M (*m/z* 327 > 291.3 and 327 > 309.40), the internal standard PGE‐M‐d6 (*m/z* 333 > 297.3 and 333 > 315.4), 11‐d‐TXB_2_ (*m/z* 367.2 > 305.1 and 367.2 > 349.2) and the internal standard 11‐d‐TXB_2_‐d4 (*m/z* 371.2 > 309.1 and 371.2 > 353.2).

PGE‐M and 11‐d‐TXB_2_ were quantified against their respective internal standard with a calibration range 0.1‐50 ng/mL. The limit of detection (LOD) for both analytes was 20 pg/mL, which was the concentration assigned to any sample with undetectable u11‐d‐TXB_2_.

The urinary creatinine (Cr) concentration in mg/ml was measured using a creatinine colorimetric assay (Cayman Chemical, Ann Arbor, MI). All uPGE‐M and u11‐d‐TXB_2_ data are expressed per mg creatinine.

Red blood cell (RBC) membrane EPA content was measured by LC‐MS/MS.^1^, ^2^ Fatty acid profiles in RBC membranes in seAFOod trial participants have been reported previously.^1^, ^2^ Data are expressed as the % of total fatty acids.

### 2.3 Statistical analysis

Baseline urinary biomarker data were not normally distributed. Therefore, data were logarithmically transformed prior to parametric univariate (*t* test/ANOVA) and multivariate regression analysis of differences in urinary values related to clinical characteristics.

The effect of the trial interventions on uPGE‐M and u11‐d‐TXB_2_ levels is reported as the percentage change at V4 or V6 from the corresponding V1 value per participant, as well as the absolute analyte concentration at individual time‐points.^7^ Comparisons between the four trial treatment groups were performed using the Chi‐squared test (for the % of participants with a reduction in biomarker level at V4 or V6 compared to V1) or the Kruskall‐Wallis test (with post hoc Bonferroni testing) for uPGE‐M and u11‐d‐TXB_2_ concentrations. Comparison of the treatment effect of EPA and aspirin, alone and in combination, at V4 and V6 was carried out with the Wilcoxon rank sum test.

The relationship between the *baseline* uPGE‐M or u11‐d‐TXB_2_ level at V1 and colorectal polyp recurrence in the placebo group only, as well as the relationship between the *on‐treatment* uPGE‐M or u11‐d‐TXB_2_ level (as the absolute value at V4 or the absolute difference between the V1 and V4 level) and colorectal polyp number after any active treatment, was investigated using negative binomial (for colorectal polyp number) or logistic (for the polyp detection rate [PDR; the % number of individuals with one or more colorectal polyps], and the presence or absence of ‘high risk’ findings [five or more colorectal polyps of any size, or two or more colorectal polyps, if at least one has ‘advanced’ characteristics {15}]) regression models. Both models compared urinary biomarker value quartiles. A low uPGE‐M or u11‐d‐TXB_2_ level was defined as being in the lowest quartile (Q1) and urinary biomarker values in Q2‐4 were classified as ‘high’ in both models. Consistent with the primary seAFOod trial analysis, repeat colonoscopy at baseline was included as a co‐variate and data were adjusted for the BCSP research site.^1^ Sex was included as a term given the consistent, strong relationship between male sex and colorectal polyp recurrence in previous colonoscopic surveillance studies.^17^, ^18^

The performance characteristics of a previously published threshold uPGE‐M value (5.34 ng/mg), which has been reported to distinguish individuals with reduced risk of ‘advanced’ polyp,^9^ for detection of one or more polyps in the seAFOod trial placebo arm was derived by bootstrapping (×1000) for derivation of a receiver‐operating characteristic curve (ROC).

All statistical analysis was performed using R studio (version 2021.09.0).

## 3 RESULTS

### 3.1 Urinary biomarkers of PGE_2_ synthesis and platelet activation in ‘high risk’ colorectal polyp patients

The distribution of urine samples collected at the three trial visits from seAFOod trial participants is described in Table S1. Baseline (pretreatment) uPGE‐M data were available for 601 (85%) and baseline (pretreatment) u11‐d‐TXB_2_ data were available for 471 (67%) trial participants. Ninety‐nine individuals did not provide a V1 sample. Seven V1 urine samples were not measured for either analyte because of a storage temperature deviation (>−20°C) at the research site. An additional 130 V1 samples had a quantifiable uPGE‐M level, but no corresponding u11‐d‐TXB_2_ value, due to absence of a clear, quantifiable chromatographic peak.

Overall, the median (interquartile range [IQR]) uPGE‐M level at entry to the seAFOod trial was 7.6 (4.5‐12.5) ng/mg (corresponding mean [SD] value 9.9 [9.9] ng/mg), with a baseline median u11‐d‐TXB_2_ level of 538 (399‐770) pg/mg (corresponding mean value 674 [561] pg/mg). Levels of uPGE‐M and u11‐d‐TXB_2_ stratified for individual clinical characteristics are described in Table 1.

**TABLE 1:** Comparison of pretreatment uPGE‐M and u11‐d‐TXB_2_ levels according to clinical characteristics of seAFOod trial participants.

	n	uPGE‐M (ng/mg Cr)^a^	Univariate *P* ^b^	Multivariate *P* ^c^	n	u11‐d‐TXB_2_ (pg/mg Cr)^a^	Univariate *P* ^b^
Overall trial population	601	7.6 (4.5‐12.5)			471	538 (399‐770)	
Sex							
Male	480	8.1 (4.9‐13.3)	<.001	<.0001	378	523 (396‐752)	.56
Female	121	5.1 (3.2‐9.0)			93	574 (412‐794)	
Body mass index (BMI)							
Underweight (<18.5 Kg/m^2^)	3	5.0 (3.7‐8.2)	.36	–	3	468 (439‐1466)	.80
Normal (≥18.5 to <25 Kg/m^2^)	106	7.5 (4.3‐12.3)			92	533 (378‐806)	
Overweight (≥25 to <30 Kg/m^2^)	263	8.1 (4.7‐13.2)			166	557 (389‐758)	
Obese (≥30 Kg/m^2^)	227	7.3 (4.2‐11.6)			209	536 (433‐759)	
Diabetes							
No	535	7.3 (4.4‐12.2)	.002	<.0001	422	528 (391‐747)	.25
Yes	66	9.8 (6.3‐15.6)			49	618 (463‐1014)	
Tobacco smoking							
Never	213	6.7 (3.9‐10.8)	.002	.005	159	490 (387‐642)	.01
Ever	293	7.6 (4.6‐12.7)			234	537 (399‐798)	
Current	95	9.1 (5.8‐14.1)			78	706 (470‐1072)	
Alcohol intake							
None	94	6.1 (3.6‐10.1)	<.001	<.001	70	494 (381‐699)	.12
1‐7 units/week	205	5.9 (3.8‐10.2)			160	536 (411‐716)	
8‐21 units/week	175	8.9 (5.4‐12.6)			141	528 (405‐835)	
≥22 units/week	125	10.1 (6.0‐16.3)			98	604 (417‐800)	

^a^ Data are presented as the median and interquartile range.

^b^ Two‐sample *t* test for binary variables or ANOVA for multiclass variables on log‐transformed data.

^c^ Variables that were statistically significant by univariate testing were taken through to a multivariate regression model. BMI and alcohol intake were not available for two participants with a PGE‐M value. The BMI was missing for one participant who had a baseline 11‐d‐TXB_2_ value and alcohol intake was missing for two participants who had a baseline 11‐d‐TXB_2_ value.

Consistent with previous reports, pretreatment uPGE‐M levels were higher in men than women.^19^ We also observed that current and former tobacco smokers had elevated baseline uPGE‐M levels compared to individuals who had never smoked.^20^ Individuals with a diagnosis of diabetes had significantly higher baseline uPGE‐M levels than nondiabetics.^7^, ^10^ We also observed a relationship between increasing alcohol intake and uPGE‐M levels (Table 1).

There was no significant difference in pretreatment u11‐d‐TXB_2_ levels between men and women in the seAFOod trial cohort of individuals, who had recently had multiple colorectal polyps removed (Table 1). Current and ex‐smokers had higher levels of u11‐d‐TXB_2_ than never smokers, in keeping with previous reports^21^, ^22^ (Table 1).

At baseline, prior to starting any trial intervention, there was a weak (*R* = .29, *P* < .0001), relationship between the uPGE‐M level and u11‐d‐TXB_2_ level in individual participants (Figure S1).

### 3.2 The effect of EPA and aspirin on uPGE‐M levels

**FIGURE 1:**
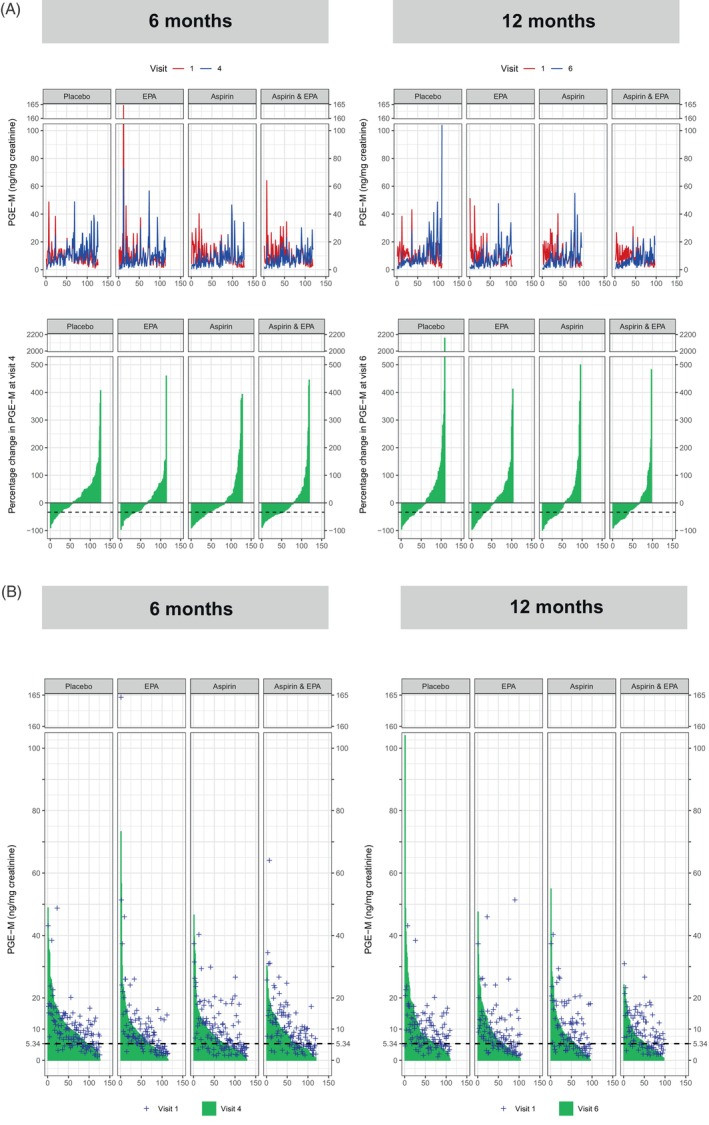
The percentage change in uPGE‐M level (A) and absolute pretreatment and on‐treatment PGE‐M levels (B) at 6 months (V4) and 12 months (V6) in seAFOod trial participants according to treatment group. The x axis denotes the number of participants in each group with paired uPGE‐M data. In (A), paired V1 and later time‐point data are presented in parallel, in order to highlight wide within‐individual variation in PGE‐M levels. Individual treatment group summary data and statistical comparisons are presented in Table S2. The dashed line in (A) denotes the 33.5% reduction threshold previously considered to be associated with reduced risk of ‘advanced’ adenoma.^10^ The dashed line in (B) denotes a uPGE‐M value of 5.34 ng/mg below which participants in the AFPPS trial had reduced risk of ‘advanced’ adenoma.^9^

The effect of EPA and aspirin treatment, alone and in combination, on uPGE‐M levels is presented as the % change from baseline (V1) (Figure 1A), as well as the uPGE‐M concentration (Figure 1B), at 6 months (V4) and 12 months (V6). In general, there was marked variability in uPGE‐M levels over time within individuals, which was apparent from the placebo arm of the seAFOod trial (Figure 1A). After treatment with EPA alone for 6 months, there was a larger proportion of trial participants with a lower uPGE‐M level at V4 than the corresponding V1 level (59%) in comparison with the placebo group (45%; Figure 1A and Table 2). The median (interquartile range [IQR]) uPGE‐M concentration at V4 in those allocated EPA was 6.9 (3.5‐10.9) ng/mg, compared to 9.1 (5.3‐13.4) ng/mg in the placebo group (*P* ≤ .01; Figure 1B and Table 2). Aspirin treatment was associated with a larger treatment effect on uPGE‐M levels than EPA with corresponding values of 66% and 6.1 (3.7‐10.2) ng/mg at V4 (*P* ≤ .05 and ≤.001, respectively, compared to placebo; Figure 1A,B and Table 2). Combination EPA and aspirin treatment was not associated with a larger treatment effect on uPGE‐M than aspirin treatment alone with 66% of individuals allocated to both agents displaying a lower V4 value than the corresponding V1 level and a median uPGE‐M concentration of 5.8 (3.9‐11.4) ng/mg (Figure 1B and Table 2). At 12 months, the treatment effect of EPA and aspirin, alone and in combination, was maintained compared to V4, with no statistically significant difference in either the % of participants with a lower uPGE‐M level compared to baseline or the uPGE‐M concentration between V4 and V6 values (Figure 1A,B and Table 2).

**TABLE 2:** Comparison of the change from the baseline (V1) value and absolute concentration of uPGE‐M and u11‐d‐TXB_2_ at V4 and V6 between seAFOod trial treatment groups.

uPGE‐M	Placebo	EPA	Aspirin	Aspirin + EPA	*P* value
V1					
Number of participants	150	152	151	148	
Median (IQR) uPGE‐M concentration (ng/mg Cr)	8.4 (5.2‐12.7)	7.4 (4.4‐12.2)	6.9 (4.0‐12.3)	7.6 (4.6‐12.4)	.42
Number (%) with uPGE‐M concentration <5.34 ng/mg Cr	39 (26)	53 (35)	57 (38)	50 (34)	.16
V4					
Number of participants	126	114	128	119	
Median (IQR) uPGE‐M concentration (ng/mg Cr)	9.1 (5.3‐13.4)	6.9 (3.5‐10.9)**	6.1 (3.7‐10.2)***	5.8 (3.9‐11.4)*	<.001^a^ ^,^ ^b^
Number (%) with any reduction in uPGE‐M	57 (45)	67 (59)	84 (66)*	79 (66)**	.002^c^
Number (%) with >33.5% reduction in uPGE‐M	25 (20)	35 (31)	44 (34)	51 (43)***	.001^c^
Number (%) with uPGE‐M concentration <5.34 ng/mg Cr	32 (25)	48 (42)	53 (41)	50 (42)*	.01^c^
V6					
Number of participants	109	103	96	97	
Median (IQR) uPGE‐M concentration (ng/mg Cr)	6.9 (4.5‐12.9)	6.1 (3.4‐11.1)	6.9 (3.3‐10.8)	5.7 (3.2‐8.7)*	.07^a^ ^,^ ^b^
Number (%) with any reduction in uPGE‐M	61 (56)	55 (53)	55 (57)	68 (70)	.08^c^
Number (%) with >33.5% reduction in uPGE‐M	36 (33)	32 (31)	42 (44)	39 (40)	.20^c^
Number (%) with uPGE‐M concentration <5.34 ng/mg Cr	39 (36)	43 (42)	38 (40)	46 (47)	.39^c^

u11‐d‐TXB_2_	Placebo	EPA	Aspirin	Aspirin + EPA	*P* value
V1					
Number of participants^d^	121	115	115	113	
Median (IQR) u11‐d‐TXB_2_ concentration (pg/mg Cr)	542 (400‐818)	559 (419‐736)	504 (388‐736)	550 (400‐782)	.45
V4					
Number of participants^e^	97	82	93	87	
Median (IQR) u11‐d‐TXB_2_ concentration (pg/mg Cr)	645 (482‐947)	515 (335‐776)*	133 (27‐266)***	132 (30‐267)***	<.001^a^ ^,^ ^b^
Number (%) with any reduction in u11‐d‐TXB_2_	37 (38)	50 (61)*	84 (90)***	80 (92)***	<.001^c^
V6					
Number of participants^e^	87	78	60	70	
Median (IQR) u11‐d‐TXB_2_ concentration (pg/mg Cr)	629 (413‐895)	567 (404‐802)	329 (198‐565)***^,¶^	272 (151‐435)***^,¶^	<.001^a^ ^,^ ^b^
Number (%) with any reduction in u11‐d‐TXB_2_	41 (47)	37 (47)	43 (72)*^,^†	58 (83)***	<.001^c^

^a^ Urinary biomarker concentrations were compared to the Kruskal‐Wallis test.

^b^ Post hoc intergroup comparisons vs the placebo group were significant at the following levels; *P ≤ .05, **P ≤ .01, ***P ≤ .001.

^c^ The number (%) of participants was compared to a Chi‐squared test.

^d^ There were seven participants with a V1 u11‐d‐TXB_2_ value, two participants with paired V1‐V4 u11‐d‐TXB_2_ values and one participant with a paired V1‐V6 u11‐d‐TXB_2_ value that had missing data on treatment allocation and were excluded.

^e^ uPGE‐M and u11‐d‐TXB_2_ levels at V1, V4 and V6 in individual treatment groups were compared using the Wilcoxon rank sum test. There was no statistically significant difference between V4 and V6 values except for the aspirin effect on u11‐d‐TXB_2_ levels at V6 compared to V4 (†denotes *P* < .01, ^¶^ *P* < .001).

In an additional analysis, we assessed changes in uPGE‐M levels based on threshold values used in previous publications.^7^, ^9^, ^10^ Firstly, we dichotomized % change values at V4 and V6 based on a reduction in uPGE‐M concentration of 33.5%, which had been used by Drew and colleagues as a cut‐off value, below which there may be reduced ‘advanced’ colorectal polyp risk based on Nurses' Health Study data.^10^ Similar treatment effects were observed at V4, with more participants allocated to combination aspirin and EPA displaying a >33.5% reduction in uPGE‐M level compared to placebo (*P* ≤ .001; Figure 1A and Table 2). However, there was no discernable change in the % number of individuals with a >33.5% reduction in uPGE‐M at V6 compared to V1 for any treatment group (Figure 1A and Table 2). Secondly, we analysed the uPGE‐M data according to a threshold uPGE‐M value of 5.34 ng/mg that has been reported to be the level below which participants had reduced risk of ‘advanced’ adenoma in the AFPPS trial.^9^ There was a larger proportion of individuals (42%) that received combination aspirin and EPA treatment who had a uPGE‐M concentration <5.34 ng/mg at V4 compared to the placebo group (25%; *P* ≤ .05) (Figure 1B and Table 2). There was no statistically significant difference from the placebo group in the % of individuals below −33.5% and 5.34 ng/mg thresholds in the three active treatment groups at V6 (Figure 1 and Table 2).

### 3.3 The effect of EPA and aspirin on u11‐d‐TXB_2_ levels

**FIGURE 2:**
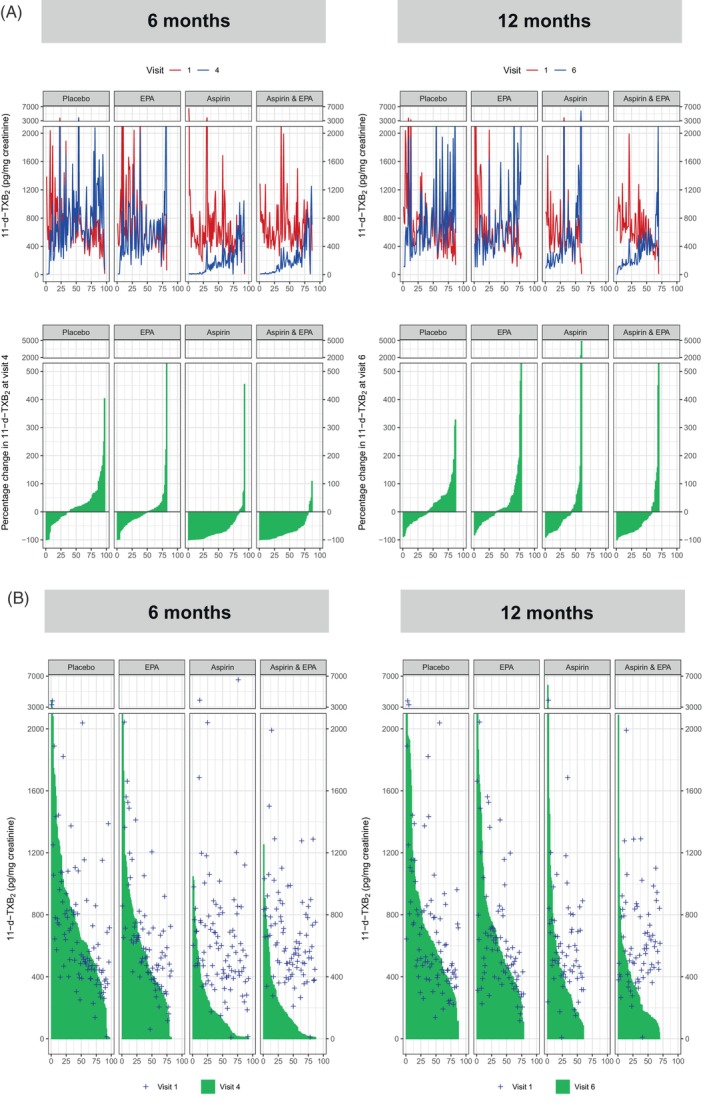
The percentage change in u11‐d‐TXB_2_ level (A) and absolute pretreatment and on‐treatment u11‐d‐TXB_2_ levels (B) at 6 months (V4) and 12 months (V6) in seAFOod trial participants according to treatment group. The x axis denotes the number of participants in each group with paired u11‐d‐TXB_2_ data. In (A), paired V1 and later time‐point data are presented in parallel, in order to highlight wide within‐individual variation in 11‐d‐TXB_2_ levels. Individual treatment group summary data and statistical comparisons are presented in Table S2.

Marked variability in u11‐d‐TXB_2_ levels over time within individuals was apparent in participants in the placebo arm of the seAFOod trial (Figure 2A). There was a modest treatment effect of EPA (approximately 8% reduction) on u11‐d‐TXB_2_ levels at V4, which was absent at V6 (Figure 2A,B and Table 2). Consistent with dose‐dependent inhibitory activity of EPA on COX‐1, there was an inverse relationship between the RBC membrane EPA content and the corresponding u11‐d‐TXB_2_ level in seAFOod trial participants who did not receive aspirin (*r* = −.27; *P* < .001), which was most pronounced in those that were randomised to active EPA (Figure S2). A similar relationship was not evident between the RBC membrane EPA level and the uPGE‐M concentration in the same trial participants (data not shown).

By contrast, there was a marked (approximately 74%) reduction in u11‐d‐TXB_2_ concentration at V4 in participants allocated to aspirin alone compared to the placebo group (Figure 2A,B and Table 2). The proportion of individuals in the aspirin group with a lower u11‐d‐TXB_2_ level at V4 compared to baseline (V1) was 90% compared to 38% in the placebo group (*P* ≤ .001; Figure 2A and Table 2). There were lower u11‐d‐TXB_2_ levels in the aspirin group (median [IQR] u11‐d‐TXB_2_ concentration 133 [27‐266] pg/mg) compared to those allocated to placebo (645 [482‐947] pg/mg) at V4 (*P* ≤ .001; Figure 2B and Table 2). There was no evidence of an additive relationship between aspirin and EPA after 6 months on‐trial in individuals randomised to combination treatment (Figure 2A,B and Table 2).

After 12 months on treatment (V6), there was a marked reduction in the effect of aspirin treatment alone (but not for EPA) on u11‐d‐TXB_2_ levels at V6 compared to the treatment effect at V4 (Figure 2A,B and Table 2). However, u11‐d‐TXB_2_ levels remained statistically significantly lower in the aspirin group at V6 (median [IQR] 329 [198‐565] pg/mg) compared to the placebo group (629 [413‐895]; *P* ≤ .001). Similar findings were apparent in the combination aspirin and EPA arm with a significant reduction in the treatment effect on u11‐d‐TXB_2_ levels at V6 compared to V4 (Figure 2A,B and Table 2).

### 3.4 The relationship between treatment effects on uPGE‐M and u11‐d‐TXB_2_ levels at participant‐level

There was evidence of a weak correlation between the change in uPGE‐M from V1 and the respective change in u11‐d‐TXB_2_ concentration from baseline in each participant at V4 and V6 (analysed as the absolute change in analyte concentration and as the % change from baseline) in each of the trial four treatment groups (Figure S3), in keeping with the shared COX‐inhibitory properties of aspirin and EPA.

### 3.5 Factors associated with reduced treatment effects on urinary biomarkers at 12 months

We postulated that the reduction in overall treatment effect on u11‐d‐TXB_2_ levels at V6 compared to V4 could be explained by reduced treatment compliance at the later time‐point. We previously reported overall capsule and tablet compliance in the seAFOod trial, which were both excellent,^1^ but we were unable to distinguish accurately between compliance levels at V4 and V6. However, trial participants were asked whether they were still taking capsules and tablets at each trial visit. Therefore, we performed a ‘per‐protocol’ analysis by excluding individuals who reported not taking capsules and/or tablets at V4 and/or V6 (Table S2 and Figure S4). There was no significant change in the effect of either EPA or aspirin on uPGE‐M or u11‐d‐TXB_2_ levels at V4 or V6 time‐points compared to the full analysis, which had included all participants who provided paired urine samples for urinary biomarker measurements (Table 2 and Figures 1 and 2). Of note, there was no diminution of the reduction in treatment effect of aspirin on u11‐d‐TXB_2_ levels at V6 compared to V4 after exclusion of those that had stopped taking trial treatment (Table S2 and Figure S4). This suggests that reduced IMP use at V6 does not completely explain the loss of the treatment effect of aspirin on u11‐d‐TXB_2_ levels over time.

We also noted that there were more participants with a large increase in u11‐d‐TXB_2_ concentration from baseline values at 12 months compared to the 6‐month data, even in the groups who were allocated to aspirin. It has previously been reported that the colonoscopy procedure is associated with immediate, but transient, bacterial endotoxaemia and an increase in plasma TXB_2_ levels that returned to baseline levels by 24 hours postprocedure in some individuals.^23^ The seAFOod trial protocol stipulated that a urine sample was collected on the same day as (after) the trial exit colonoscopy (the day after the final IMP doses). However, a few participants submitted a urine sample three or more days after the colonoscopy for practical reasons, which gave us an opportunity to investigate whether the proximity to colonoscopy was associated with higher u11‐d‐TXB_2_ levels at V6 in some individuals. The u11‐d‐TXB_2_ concentration in urine samples provided by participants in the placebo group on the same day as the colonoscopy (median [IQR] 617 [431‐857] pg/mg) was higher than the u11‐d‐TXB_2_ levels in urine samples provided ≥3 days after the colonoscopy from individuals from all treatment groups, who last took IMP at least 4 days previously (428 [309‐555] pg/mg; *P* = .01; Mann‐Whitney *U* test; Figure S5A). However, there was no significant difference in uPGE‐M concentration in V6 samples collected on the same day as (7.1 [4.6‐11.4] ng/mg) vs ≥3 days after colonoscopy (7.7 (6.1‐9.0]; *P* = .41; Student's *t* test on log‐transformed data; Figure S5B). This suggests that the proximity to a colonoscopy of the V6 sample collection may contribute to the difference between u11‐d‐TXB_2_ levels observed at V6 compared to V4 even in those allocated to aspirin, possibly reflecting increased nonplatelet TXA_2_ synthesis associated with the colonoscopy procedure and/or the bowel preparation. Given the reduction in treatment effect of EPA and aspirin on urinary analyte levels at V6 compared to V4 (noting that last IMP dosing was the day before V6 urine sampling) and the confounding effect of the proximity to colonoscopy, we restricted further analyses of the predictive value of uPGE‐M and u11‐d‐TXB_2_ levels on colorectal polyp outcomes in the seAFOod trial to urinary analyte values measured at 6 months (V4).

### 3.6 The relationship between uPGE‐M and u11‐d‐TXB_2_ levels with colorectal polyp outcomes

Analysis of colorectal polyp outcomes in seAFOod trial participants, who were allocated to placebos only, allowed us to investigate whether urinary biomarker levels at trial entry (V1) were predictive of colorectal polyp outcomes 12 months after clearance colonoscopy, independently of any intervention.

The baseline uPGE‐M value was not associated with an increased colorectal polyp number or PDR in seAFOod trial participants allocated to placebos only (Table 3). There were only a small number of participants with ‘high risk’ polyp findings in each baseline uPGE‐M quartile limiting interpretation of this endpoint (Table 3). There was no difference in total colorectal polyp risk in placebo group participants according to the threshold uPGE‐M value of 5.34 ng/mg (Table S3A), below which risk of ‘advanced’ adenoma was lower in the AFPPS trial.^9^ The sensitivity and specificity of the 5.34 ng/mg cut‐off for all colorectal polyp detection was 92% and 9%, respectively (area under ROC 0.51; positive predictive value 62%). There were only 4 advanced colorectal polyps found in individuals randomised to placebos only in the seAFOod trial so that a meaningful analysis of advanced polyp prediction was not possible.

**TABLE 3:** The relationship between the baseline urinary biomarker value and colorectal polyp outcomes at seAFOod trial exit colonoscopy in participants randomised to placebos only.

	Colorectal polyp number		PDR (% of individuals with one or more polyps)		‘High risk’ findings	
	n	IRR^a^ (95% CI)^b^	Cases/total (n)	Odds ratio (95% CI)^b^	Cases/total (n)	Odds ratio (95% CI)^b^
PGE‐M (ng/mg creatinine)						
Q1 (0.45‐4.52)	25	Reference	17/25	Reference	1/25	Reference
Q2 (4.53‐7.67)	34	1.17 (0.44, 3.17)	20/34	0.70 (0.24, 2.04)	4/33	3.43 (0.37, 31.61)
Q3 (7.68‐12.63)	39	1.08 (0.41, 2.88)	22/39	0.67 (0.31, 1.44)	2/39	1.19 (0.09, 16.11)
Q4 (12.64‐164.65)	35	2.59 (0.95, 7.05)	23/35	1.03 (0.37, 2.83)	4/32	3.40 (0.32, 35.56)
11‐d‐TXB_2_ (pg/mg creatinine)						
Q1 (9‐397)	29	Reference	14/29	Reference	1/28	Reference
Q2 (398‐530)	28	**3.18 (1.54, 6.56)**	22/28	**3.56 (1.09, 11.63)**	2/26	2.10 (0.25, 17.97)
Q3 (531‐769)	19	**2.32 (1.03, 5.21)**	14/19	2.66 (0.69, 10.30)	0/18	0 (0, 0)
Q4 (770‐6530)	30	**2.26 (1.11, 4.58)**	18/30	1.78 (0.72, 4.43)	3/28	3.16 (0.36, 27.94)

*Note*: Bold signifies statistically significant values.

^a^ Incidence rate ratio.

^b^ Regression models were adjusted for sex and repeat colonoscopy at baseline.

By contrast, in comparison with seAFOod trial participants with the lowest quartile baseline u11‐d‐TXB_2_ levels, individuals with a higher baseline u11‐d‐TXB_2_ level (Q2‐4) had an increased number of polyps at subsequent colonoscopy (incidence rate ratio [IRR] [95% confidence interval (CI)] 2.26 [1.11, 4.58] for Q4) and also a higher PDR, which reached statistical significance for Q2 (odds ratio [OR] [95% CI] 3.56 [1.09, 11.63]; Table 3).

Next, we investigated the ability of the on‐treatment (V4) urinary biomarker level to predict colorectal polyp number in seAFOod trial participants in each of the three active intervention groups. We defined a ‘low’ urinary biomarker level as any value less than or equal to the Q1 value, as opposed to concentrations in Q2‐4, which were defined as ‘high’. The on‐treatment uPGE‐M concentration did not predict colorectal polyp number for any of the three active treatment groups compared to placebo, although, in all three active treatment groups, individuals with a low uPGE‐M level after 6 months' treatment had a lower, but not statistically significant, IRR value than participants with high uPGE‐M during treatment (Table 4).

**TABLE 4:** The relationship between the on‐treatment urinary biomarker level and total colorectal polyp number after 12 months of treatment with EPA and aspirin, alone or in combination, during the seAFOod polyp prevention trial.

	Placebo^a^	EPA			Aspirin			Aspirin + EPA		
PGE‐M	n	n	IRR^b^ (95% CI)^c^	*P*	n	IRR^b^ (95% CI)^c^	*P*	n	IRR^b^ (95% CI)^c^	*P*
All participants (n = 435)	114	100	0.74 (0.47, 1.17)	.20	113	0.89 (0.57, 1.38)	.59	108	1.03 (0.66, 1.62)	.89
Low PGE‐M (<4.12)^d^ (n = 107)	17	31	0.38 (0.12, 1.17)	.10	30	0.39 (0.13, 1.20)	.10	29	0.40 (0.13, 1.23)	.11
High PGE‐M (≥4.12)^d^ (n = 328)	97	69	0.81 (0.49, 1.35)	.43	83	1.04 (0.64, 1.69)	.87	79	1.30 (0.80, 2.12)	.30

	Placebo^a^	EPA			Aspirin			Aspirin + EPA		
11‐d‐TXB_2_	n	n	IRR^b^ (95% CI)^c^	*P*	n	IRR^b^ (95% CI)^c^	*P*	n	IRR^b^ (95% CI)^c^	*P*
All participants (n = 316)	87	71	1.25 (0.77, 2.02)	.37	77	0.83 (0.52, 1.33)	.45	81	**0.58 (0.36, 0.92)**	**.02**
Low 11‐d‐TXB_2_ (<107)^d^ (n = 84)	6	4	0.27 (0.06, 1.16)	.08	37	0.39 (0.14, 1.08)	.07	37	**0.34 (0.12, 0.93)**	**.04**
High 11‐d‐TXB_2_ (≥107)^d^ (n = 232)	81	67	1.37 (0.81, 2.33)	.24	40	1.08 (0.59, 2.01)	.80	44	0.61 (0.34, 1.11)	.11

^a^ Reference group.

^b^ Incidence rate ratio.

^c^ The regression model was adjusted for sex and repeat colonoscopy at baseline.

^d^ Low PGE‐M/11‐d‐TXB_2_ = quartile 1; High PGE‐M/11‐d‐TXB_2_ = quartile 2, 3 and 4 of V4 (6 months on treatment) values.

For patients who had a low u11‐d‐TXB_2_ level at 6 months (V4), all three active treatments led to a decrease in the number of colorectal polyps at colonoscopy at 12 months compared to placebo (Table 4 and Figure S6). This reduction was statistically significant in patients who received a combination of aspirin and EPA (IRR 0.34 [0.12, 0.93]). In contrast, for individuals with a high u11‐d‐TXB_2_ level at V4 during active treatment, there was no reduction in colorectal polyp number at trial exit colonoscopy (Table 4 and Figure S6).

We also explored whether the change in urinary biomarker level from baseline during treatment was predictive of colorectal polyp outcomes (Table S4). The change in uPGE‐M level at V4 from V1 during active treatment did not predict colorectal polyp number (Table S4). There was no difference in colorectal polyp risk in the active treatment groups stratified using the 33.5% reduction threshold identified by the Nurses' Health as being associated with reduced ‘advanced’ colorectal polyp risk (Table S3B).^10^

However, the highest quartile of difference between V1 and V4 u11‐d‐TXB_2_ levels (effectively those individuals with an increase in u11‐d‐TXB_2_ level during active treatment in the seAFOod trial; Table S4) was associated with increased colorectal polyp number compared to those participants with the lowest quartile u11‐d‐TXB_2_ level change (equivalent to the largest on‐treatment reduction). This relationship only attained statistical significance for analysis of the three treatment groups combined due to the small size of the higher quartiles of u11‐d‐TXB_2_ level differences in the individual active treatment groups (Table S4).

## 4 DISCUSSION

In a secondary analysis of the seAFOod polyp prevention trial, we report that aspirin 300 mg daily and, to a lesser extent, EPA 2000 mg daily both reduce levels of the stable urinary prostanoid metabolites PGE‐M and 11‐d‐TXB_2_, during dosing for up to 12 months, compatible with in vivo COX‐1 and COX‐2 inhibition. Importantly, we demonstrate that the u11‐d‐TXB_2_ level predicts subsequent colorectal polyp development independently of any chemoprevention, compatible with an emerging link between platelet activation and colorectal carcinogenesis.^24^ Another key finding is that u11‐d‐TXB_2_ suppression during treatment with aspirin and EPA predicts their chemoprevention efficacy, as measured by colorectal polyp recurrence.

Pretreatment levels of uPGE‐M in seAFOod trial participants were comparable to concentrations reported in similar populations undergoing colonoscopy and polypectomy.^7^, ^8^, ^9^ The relationship between male sex, tobacco smoking (current and previous), BMI and elevated uPGE‐M levels in the seAFOod trial population is consistent with the existing literature.^10^, ^19^, ^20^

Baseline levels of u11‐d‐TXB_2_ were higher than levels detected in AFPPS participants (which were approximately 200‐300 pg/mg creatinine^9^), but lower than those reported in the ASPIRED trial of individuals who had undergone colorectal polyp clearance in the past 9 months (median 1700 pg/mg creatinine).^7^ Consistent with AFPPS findings and several studies of individuals with cardiovascular risk factors, we also noted higher u11‐d‐TXB_2_ levels in current smokers compared to nonsmokers.^25^, ^26^ However, we did not demonstrate a sex‐difference in u11‐d‐TXB_2_ levels, unlike AFPPS.^9^ Notwithstanding methodological differences in measurement of stable urinary TXB_2_ metabolites and differences in demographics between different studies, our data support the assertion that platelet activation (as measured by u11‐d‐TXB_2_ levels) in individuals, who have developed one or more colorectal polyps and then undergone clearance colonoscopy, is similar to levels detected in populations which have similar cardiovascular/cancer risk factors such as obesity and diabetes.^27^ There are limited data on u11‐d‐TXB_2_ levels in CRC patients, but a small study (n = 10) concluded that u11‐d‐TXB_2_ excretion in CRC patients (median 1000 pg/mg creatinine) was significantly higher than matched controls that had similar clinical characteristics to the seAFOod trial population.^14^ Comparison with the median (IQR) u11‐d‐TXB_2_ level of 1060 (667‐1558) pg/mg in 192 Add‐Aspirin trial participants, who had undergone surgical resection of a stage II or III CRC, suggests that colorectal polyp patients display lower u11‐d‐TXB_2_ levels than CRC patients even after primary CRC therapy.^28^

The effect of long‐term (12 months) EPA and aspirin treatment on uPGE‐M excretion was modest, on a background of wide variability in uPGE‐M levels over time within individuals. A small (<10%) reduction in uPGE‐M concentration during treatment with a mixed omega‐3 PUFA formulation (1395 mg EPA and 1125 mg docosahexaenoic acid [DHA] daily) for 6 months in 70 patients with a history of colorectal polyps (the Tennessee Colorectal Polyp Study [TCPS]) has been reported previously.^8^ Our data also concur with the effect of aspirin 81 mg (n = 57) or 325 mg (n = 54) daily vs placebo (n = 58) on uPGE‐M levels at a single 3 month time‐point in the ASPIRED trial.^7^ In the AFPPS, on‐treatment urine samples were obtained at variable times during the third intervention year and revealed a similar reduction in uPGE‐M concentration in individuals randomised to aspirin 81 mg or 325 mg daily compared to our seAFOod trial data.^9^ In seAFOod trial participants, the treatment effect of EPA and aspirin on uPGE‐M levels was maintained up to 12 months, unlike the TCPS trial, which demonstrated a diminution of the treatment effect of mixed omega‐3 PUFAs on uPGE‐M levels at 6 months compared to 3 months, despite maintenance of high RBC omega‐3 PUFA levels.^8^

The effect of aspirin 300 mg daily on u11‐d‐TXB_2_ excretion in individuals with a history of multiple colorectal polyps adds to the substantial literature on u11‐d‐TXB_2_ as a biomarker of platelet inhibition by aspirin.^27^ The effect size of aspirin 300 mg daily on u11‐d‐TXB_2_ levels (a 74% reduction) in seAFOod trial participants was similar to that observed during treatment with aspirin 100 and 300 mg daily for at least 3 months in Add‐Aspirin trial participants.^28^ The precise contribution of platelet‐ and nonplatelet‐dependent TXB_2_ synthesis in seAFOod trial participants is unclear. However, the highly variable u11‐d‐TXB_2_ levels detected in individuals, who were randomised to active aspirin (acknowledging likely variable treatment compliance), argues that there is a significant contribution to TXB_2_ synthesis from nonplatelet sources, in which COX isoform inhibition by aspirin is incomplete, as reported by Lanas et al in familial adenomatous polyposis patients.^29^ The reduction in u11‐d‐TXB_2_ suppression at 12 months compared to 6 months in aspirin users is most likely to be due to reduced tablet compliance over time on trial but the effect of stopping IMP the day before urine and blood sampling, as per seAFOod trial protocol, is also likely to be a contributing factor.

Our data are consistent with the modest antiplatelet activity of EPA.^30^ Importantly, the level of tissue EPA incorporation (measured by RBC membrane levels) was related to the u11‐d‐TXB_2_ level, particularly in EPA users. The relationship between dietary omega‐3 PUFA intake, tissue omega‐3 PUFA levels and u11‐d‐TXB_2_ excretion should be explored further in order to delineate the link between dietary omega‐3 PUFA intake and platelet activation. The absence of an additive relationship between aspirin and EPA on u11‐d‐TXB_2_ levels in seAFOod trial participants that were randomised to both active treatments is consistent with several platelet function studies [reviewed in 30].

A strength of our study was the ability to test the predictive value of uPGE‐M and u11‐d‐TXB_2_ for determining subsequent colorectal polyp risk in the placebo group, as well as for testing the utility of on‐treatment urinary biomarker levels for therapeutic response prediction, using seAFOod trial colonoscopy outcomes.

There was no statistically significant relationship between the baseline uPGE‐M level and colorectal polyp outcomes 12 months later. However, a higher baseline u11‐d‐TXB_2_ level did predict increased colorectal polyp risk (as measured by colorectal polyp number) at seAFOod trial exit colonoscopy 12 months later compared to individuals with a low (Q1) u11‐d‐TXB_2_ level. The AFPPS reported null findings for the relationship between both uPGE‐M and u11‐d‐TXB_2_ levels (measured after 3 years on‐trial) and colorectal polyp occurrence (reported as the PDR) in the placebo group; however, colorectal polyp number outcomes were not described.^9^ Using a similar approach to stratify individuals as having a high vs low u11‐d‐TXB_2_ level, Rade and colleagues have reported that a high u11‐d‐TXB_2_ level predicts all‐cancer mortality, irrespective of aspirin use, in a large longitudinal cohort study.^26^ A prospective validation study of the relationship between the u11‐d‐TXB_2_ level and subsequent colorectal polyp outcomes is now required to determine the role of the baseline u11‐d‐TXB_2_ concentration as a risk biomarker.

When we investigated the relationship between the on‐treatment urinary biomarker value (at 6 months) and subsequent colorectal polyp number detected at colonoscopy, we observed a consistent reduction in colorectal polyp risk in individuals with a low uPGE‐M and u11‐d‐TXB_2_ level compared to those with higher uPGE‐M and u11‐d‐TXB_2_ values. This relationship was statistically significant only for individuals randomised to combination aspirin and EPA, which was the treatment group that displayed the largest reduction in colorectal polyp risk in the secondary individual treatment group analysis of the seAFOod trial.^1^, ^4^ There was no relationship between the on‐treatment u11‐d‐TXB_2_ level and colorectal polyp outcomes in the secondary AFPPS analysis.^9^ However, the timing of the urine sampling (in relation to the colonoscopic outcomes) was variable in AFPPS and no data were provided about treatment compliance at the time of sampling.^9^

We also show that those participants who displayed an increase in u11‐d‐TXB_2_, despite randomisation to an active treatment arm (likely due to poor treatment compliance), had increased colorectal polyp risk consistent with absence of the antineoplastic activity of aspirin. A prospective study of u11‐d‐TXB_2_ levels in patients before and during aspirin treatment in future polyp prevention trials should be considered in order to strengthen evidence that the absolute level or change from the baseline value of u11‐d‐TXB_2_ predicts colorectal polyp (and CRC) prevention by aspirin, as well as delineate clinically meaningful cut‐off values for predictive models.

Another important observation from our study is that u11‐d‐TXB_2_ levels, in particular, were affected by the proximity of the urine sampling to a colonoscopy. A plausible hypothesis is that either bowel preparation, the colonoscopy itself, or both, lead to increased prostanoid synthesis based on the data on increased plasma TXB_2_ levels during and immediately after colonoscopy from Berger et al.^23^ Our observation that plasma 15‐hydroxyeicosatetraenoic (HETE) acid (a C20:4*n*‐6 arachidonic acid metabolite) levels are increased in blood samples, which were taken at the same time as the urine sample at V6, compared to V4 samples, is also compatible with provocation of COX‐dependent prostanoid synthesis during colonoscopy.^31^

Importantly, although urine samples were stored for several years at −80°C in the seAFOod trial biobank before analysis, both urinary metabolites are considered stable over a wide temperature range and for long periods of time.^32^, ^33^ Therefore, we do not consider degradation of analytes to have contributed to these findings.

Limitations of this secondary biomarker analysis of the seAFOod trial include incomplete coverage of the original trial cohort, leading to small group sizes for some treatment group analyses. In turn, there are wide confidence intervals around some point estimates for the relationships between urinary biomarker values and colorectal polyp outcomes in the individual trial treatment groups that should be interpreted with caution.

We were, however, able to leverage trial data on treatment compliance to complete sensitivity analysis of a per‐protocol population and explore the contribution of treatment cessation to changes of the urinary biomarker data.

In summary, we report the effects of aspirin and EPA on urinary biomarkers of COX‐1 and COX‐2 activity in individuals with a history of multiple colorectal polyps taking part in the seAFOod polyp prevention trial. Urinary PGE‐M levels are highly variable over time and do not appear to have utility for colorectal polyp risk or therapeutic response prediction for aspirin or EPA. However, we suggest that u11‐d‐TXB_2_ should be further investigated as a potential biomarker of colorectal polyp risk, with which to guide a precision approach to aspirin use for cancer chemoprevention.

## AUTHOR CONTRIBUTIONS

The work reported in the paper has been performed by the authors, unless clearly specified in the text. Paul M. Loadman and Mark A. Hull conceived and designed the study. Mark A. Hull and Amy Downing gained approvals for the study. Ge Sun, Harriett Fuller, Hayley Fenton, Amanda D. Race, Amy Downing, Paul M. Loadman and Mark A. Hull were involved in the acquisition, analysis, or interpretation of data. Drafting of the manuscript was done by Ge Sun, Harriett Fuller, Amy Downing and Mark A. Hull. Elizabeth A. Williams, Colin J. Rees, Louise C. Brown, Paul M. Loadman and Mark A. Hull obtained funding for the study. All the authors contributed to the critical review and final approval of the manuscript. Ge Sun, Harriett Fuller, Amy Downing and Mark A. Hull have accessed and verified the underlying data. All authors were responsible for the decision to submit the manuscript.

## FUNDING INFORMATION

This project (NIHR128210) was funded by the Efficacy and Mechanism Evaluation (EME) Programme, an MRC and NIHR partnership. The views expressed in this publication are those of the authors and not necessarily those of the MRC, NIHR or the Department of Health and Social Care. MAH and CJR are NIHR Senior Investigators. Amy Downing and Mark A. Hull are supported by Cancer Research UK grant C23434/A24939.

## CONFLICT OF INTEREST STATEMENT

The authors declare no conflicts of interest.

## ETHICS STATEMENT

Our study is part of a wider programme of investigations using the seAFOod trial biobank and posttrial BCSP colonoscopy outcomes called STOP‐ADENOMA (ISRCTN05926847). Ethical approval for our study was granted by London and Surrey Borders Research Ethics Committee (19/LO/1655). All participants of the seAFOod trial provided written informed consent to collect data on posttrial BCSP outcomes and to use urine and blood samples for laboratory studies.

## Supporting information


**Data S1.** Supporting Information.

## Data Availability

The data that support the findings of our study are available from the corresponding author upon reasonable request.
