# Supplementary material for: The effect of aspirin and eicosapentaenoic acid on urinary biomarkers of prostaglandin E2 synthesis and platelet activation in participants of the seAFOod polyp prevention trial
Source: Int J Cancer. 2023 Oct 19;154(5):873–85. doi: 10.1002/ijc.34764 (PMC10952676; doi:10.1002/ijc.34764)
Supplement: Supplementary file 1 — Data S1. Supporting Information. [file IJC-154-873-s001.pdf]

The effect of aspirin and eicosapentaenoic acid on urinary biomarkers of prostaglandin E_2 synthesis and platelet activation in participants of the seAFOod polyp prevention trial

Ge Sun, Harriett Fuller, Hayley Fenton**,** Amanda D Race**,** Amy Downing**,** Elizabeth A Williams, Colin J Rees, Louise C Brown, Paul M Loadman**,** Mark A Hull

(Bold denotes joint first Author)

Supplementary Material – table of contents

Supplementary Table 1

Supplementary Table 2

Supplementary Table 3A

Supplementary Table 3B

Supplementary Table 4

Supplementary Figure 1

Supplementary Figure 2

Supplementary Figure 3

Supplementary Figure 4

Supplementary Figure 5

Supplementary Figure 6

Supplementary Table 1. Number of seAFOod trial participants with uPGE-M and u11-d-TXB_2 data according to the profile of urine sample measurements

Trial Visits†	n with uPGE-M data	n with u11-d-TXB_2 data
V1, V4, V6	378	269
V1, V4	109	92
V1, V6	27	27
V4, V6	0	0
V1 only	87	83
V4 only	0	0
V6 only	11	108

† V1, baseline (pre-treatment); V4, 6 months (mid-treatment); V6, 12 months (end of treatment.

uPGE-M	Placebo	EPA	Aspirin	Aspirin + EPA	P value
V4					
Number of participants	126	109	119	108	
Median (IQR) uPGE-M concentration (ng/mg Cr)	9.1 (5.3-13.4)	6.9 (3.5-10.6)**	6.1 (3.7-10.0)***	5.5 (3.9-10.4)**	<0.001[1-2]
Number (%) with any reduction in uPGE-M	57 (45)	65 (60)	80 (67) **	72 (67) **	0.001[3]
Number (%) with >33.5% reduction in uPGE-M	25 (20)	34 (31)	42 (35)	46 (43)**	0.002[3]
Number (%) with uPGE-M concentration <5.34 ng/mg Cr	32 (25)	46 (42)	50 (42)	48 (44)*	0.007[3]
V6					
Number of participants	109	85	81	88	
Median (IQR) uPGE-M concentration (ng/mg Cr)	6.9 (4.5-12.9)	6.1 (3.4-10.9)	7.0 (3.1-10.7)	5.7 (3.2-8.6)	0.06[1-2]
Number (%) with any reduction in uPGE-M	61 (56)	46 (54)	48 (59)	62 (70)	0.11[3]
Number (%) with >33.5% reduction in uPGE-M	36 (33)	27 (32)	35 (43)	35 (40)	0.34[3]
Number (%) with uPGE-M concentration <5.34 ng/mg Cr	39 (36)	37 (44)	33 (41)	41 (47)	0.46[3]

u11-d-TXB2	Placebo	EPA	Aspirin	Aspirin + EPA	P value
V4					
Number of participants[4]	97	79	85	78	
Median (IQR) u11-d-TXB_2 concentration (pg/mg Cr)	645 (482-947)	498 (330-773) **	125 (27-233) ***	113 (27-214)***	< 0.001[1-2]
Number (%) with any reduction in u11-d-TXB_2	37 (38)	48 (61)*	79 (93)***	75 (96)***	< 0.001[3]
V6					
Number of participants[4]	87	64	51	62	
Median (IQR) u11-d-TXB_2 concentration (pg/mg Cr)	629 (413-895)	562 (420-825)	317 (198-548)*** ¶	218 (140-378)*** ¶	< 0.001[1-2]
Number (%) with any reduction in u11-d-TXB_2	41 (47)	32 (50)	38 (75)*†	53 (85)***	< 0.001[3]

Supplementary Table 2. Comparison of the change from baseline (V1) value and absolute concentration of uPGE-M and u11-d-TXB_2 at V4 and V6 between seAFOod trial treatment groups, excluding participants reported to be not taking IMP at V4 and/or V6. [1]Urinary biomarker concentrations were compared with the Kruskal-Wallis test. [2]Post-hoc inter-group comparisons *versus* the placebo group were significant at the following levels; *P≤0.05, **P≤0.01, ***P≤0.001. [3]The number (%) of participants was compared with a Chi-squared test. [4]There were two participants with paired V1-V4 u11-d-TXB_2 values and one participant with paired V1-V6 u11-d-TXB_2 values that had missing data on treatment allocation and were excluded. uPGE-M and u11-d-TXB_2 levels at V4 and V6 in individual treatment groups were compared using the Wilcoxon

rank sum test. There was no statistically significant difference between V4 and V6 values except for the aspirin effect on u11-d-TXB_2 levels at V6 compared with V4 († denotes $P<0.01$, ¶ $P<0.001$).

Supplementary Table 3A. Colorectal polyp outcomes according to a threshold baseline uPGE-M value of 5.34 ng/mg in individuals receiving placebos only in the seAFOod trial.

	**Colorectal polyp number**		**PDR (% of individuals with one or more polyps)**		**'high risk' findings**	
	n	IRR* (95% CI)**	cases/total (n)	Odds ratio (95% CI)**	cases/total (n)	Odds ratio (95% CI)**
**PGE-M (ng/mg creatinine)**						
<5.34† (n=34)	34	0.75 (0.35-1.63)	22/34	1.14 (0.61-2.13)	2/33	0.70 (0.15-3.25)
≥5.34† (n=99)	99	reference	60/99	reference	9/96	reference

Supplementary Table 3B. Colorectal polyp number according to a threshold 33.5% reduction in uPGE-M level from baseline in individuals receiving active treatment in the seAFOod trial.

**PGE-M**	**Placebo‡**	**EPA**			**Aspirin**			**Aspirin + EPA**		
	n	n	IRR* (95% CI)**	p	n	IRR* (95% CI)**	p	n	IRR* (95% CI)**	p
All participants (n=435)	114	100	0.74(0.47-1.17)	0.20	113	0.89(0.57-1.38)	0.59	108	1.03(0.66-1.62)	0.89
<33.5% reduction (n=300)	91	72	0.69(0.44-1.09)	0.11	76	1.09(0.70-1.70)	0.71	61	1.14(0.71-1.84)	0.59
≥33.5% reduction (n=135)	23	28	0.69(0.22-2.21)	0.53	37	0.42(0.14-1.25)	0.12	47	0.57(0.20-1.64)	0.30

*Incidence rate ratio

**regression models were adjusted for sex and repeat colonoscopy at baseline

†6 months on-treatment value

‡reference group

Supplementary Table 4. The relationship between the change in urinary biomarker level from baseline (V1) after 6 months treatment (V4)* and colorectal polyp number

PGE-M	Polyp number	
	n	IRR† (95% CI)
**All active treatment groups**		
Q1 (-1.34 to -0.22)	89	reference
Q2 (-0.22 to -0.06)	85	0.66 (0.42-1.05)
Q3 (-0.06 to 0.12)	80	0.84 (0.53-1.35)
Q4 (0.12 to 0.75)	67	0.64 (0.39-1.05)
**EPA**		
Q1 (-1.34 to -0.22)	22	reference
Q2 (-0.22 to -0.06)	26	0.56 (0.25-1.26)
Q3 (-0.06 to 0.12)	25	0.34 (0.15-0.77)
Q4 (0.12 to 0.75)	27	0.41 (0.18-0.91)
**Aspirin**		
Q1 (-1.34 to -0.22)	30	reference
Q2 (-0.22 to -0.06)	31	0.65 (0.32-1.32)
Q3 (-0.06 to 0.12)	30	1.15 (0.56-2.39)
Q4 (0.12 to 0.75)	22	1.28 (0.59-2.78)
**Aspirin + EPA**		
Q1 (-1.34 to -0.22)	37	reference
Q2 (-0.22 to -0.06)	28	0.79 (0.35-1.80)
Q3 (-0.06 to 0.12)	25	1.58 (0.68-3.66)
Q4 (0.12 to 0.75)	18	0.53 (0.21-1.35)

11-d-TXB_2	Polyp number	
	n	IRR† (95% CI)
**All active treatment groups**		
Q1 (-6.05 to -1.61)	75	reference
Q2 (-1.61 to -0.48)	69	1.32 (0.79-2.20)
Q3 (-0.48 to -0.07)	56	1.63 (0.95-2.81)
Q4 (-0.07 to 1.96)	29	**2.00 (1.02-3.92)**
**EPA**		
Q1 (-6.05 to -1.61)	4	reference
Q2 (-1.61 to -0.48)	11	3.04 (0.28-32.73)
Q3 (-0.48 to -0.07)	38	2.80 (0.33-24.20)
Q4 (-0.07 to 1.96)	18	3.65 (0.37-35.98)
**Aspirin**		
Q1 (-6.05 to -1.61)	34	reference
Q2 (-1.61 to -0.48)	25	1.55 (0.76-3.15)
Q3 (-0.48 to -0.07)	12	1.19 (0.48-2.96)
Q4 (-0.07 to 1.96)	6	2.07 (0.62-6.86)
**Aspirin + EPA**		
Q1 (-6.05 to -1.61)	36	reference
Q2 (-1.61 to -0.48)	32	1.01 (0.62-1.65)
Q3 (-0.48 to -0.07)	6	0.95 (0.39-2.28)
Q4 (-0.07 to 1.96)	4	1.10 (0.38-3.17)

*due to the high variability in urinary biomarker values over time, the difference between values at V1 and V4 in each individual is reported as the logV4 value minus the logV1 value.
All models were adjusted for sex and repeat colonoscopy.
†Incidence rate ratio

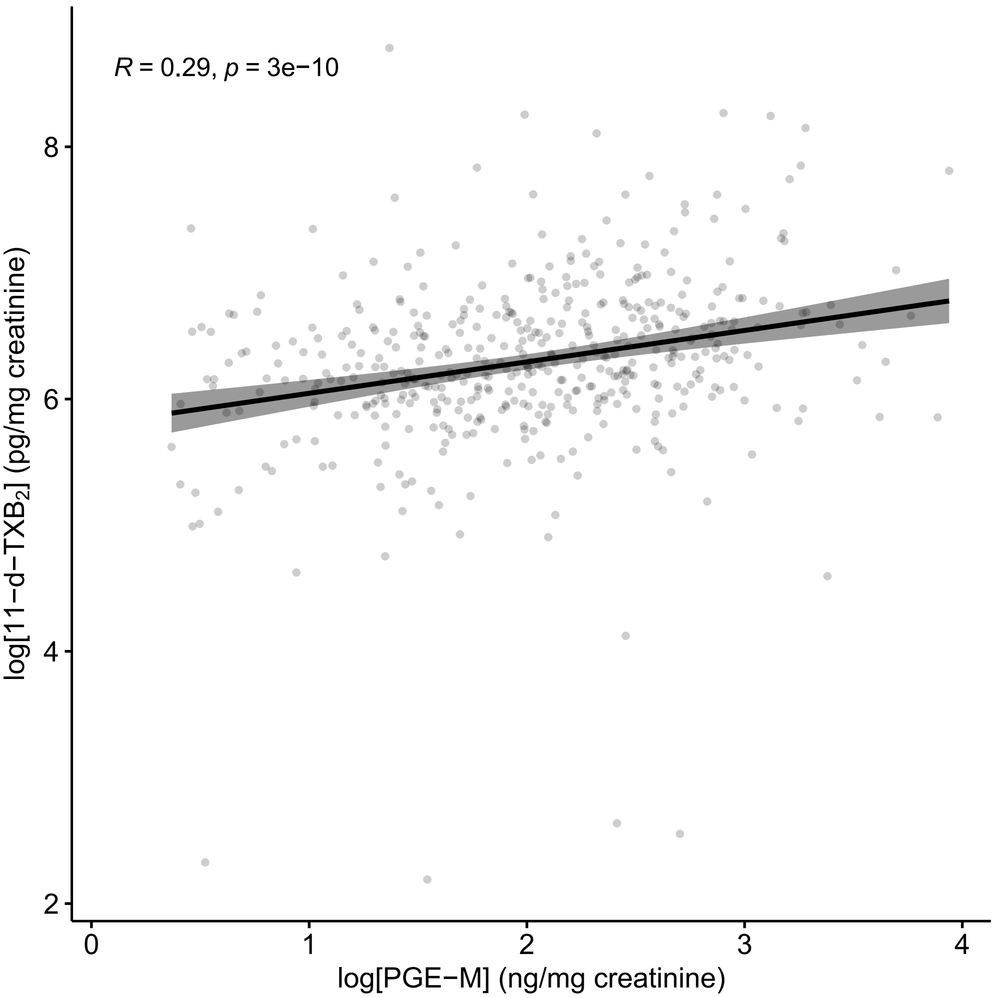

Supplementary Figure 1. Relationship between the uPGE-M and u11-d-TXB_2 concentration in seAFOod trial participants at baseline (V1). Data are presented as $\log_e$ values. Spearman's test was used to investigate the relationship between paired urinary biomarker values.

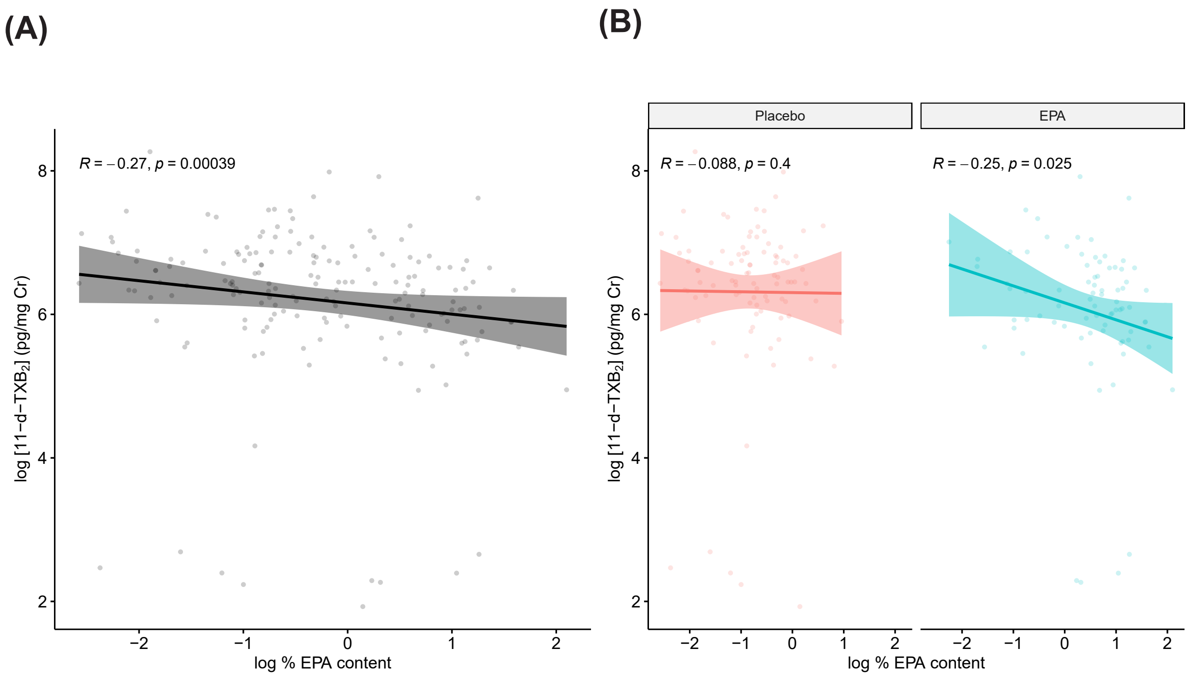

Supplementary Figure 2. The relationship between the red blood cell membrane EPA level (as % total fatty acids) and u11-d-TXB_2 concentration at V4 (after treatment for 6 months) for A) all seAFOod trial participants not randomised to aspirin, and B) participants receiving either placebos alone or EPA only. Data are presented as $\log_e$ values.

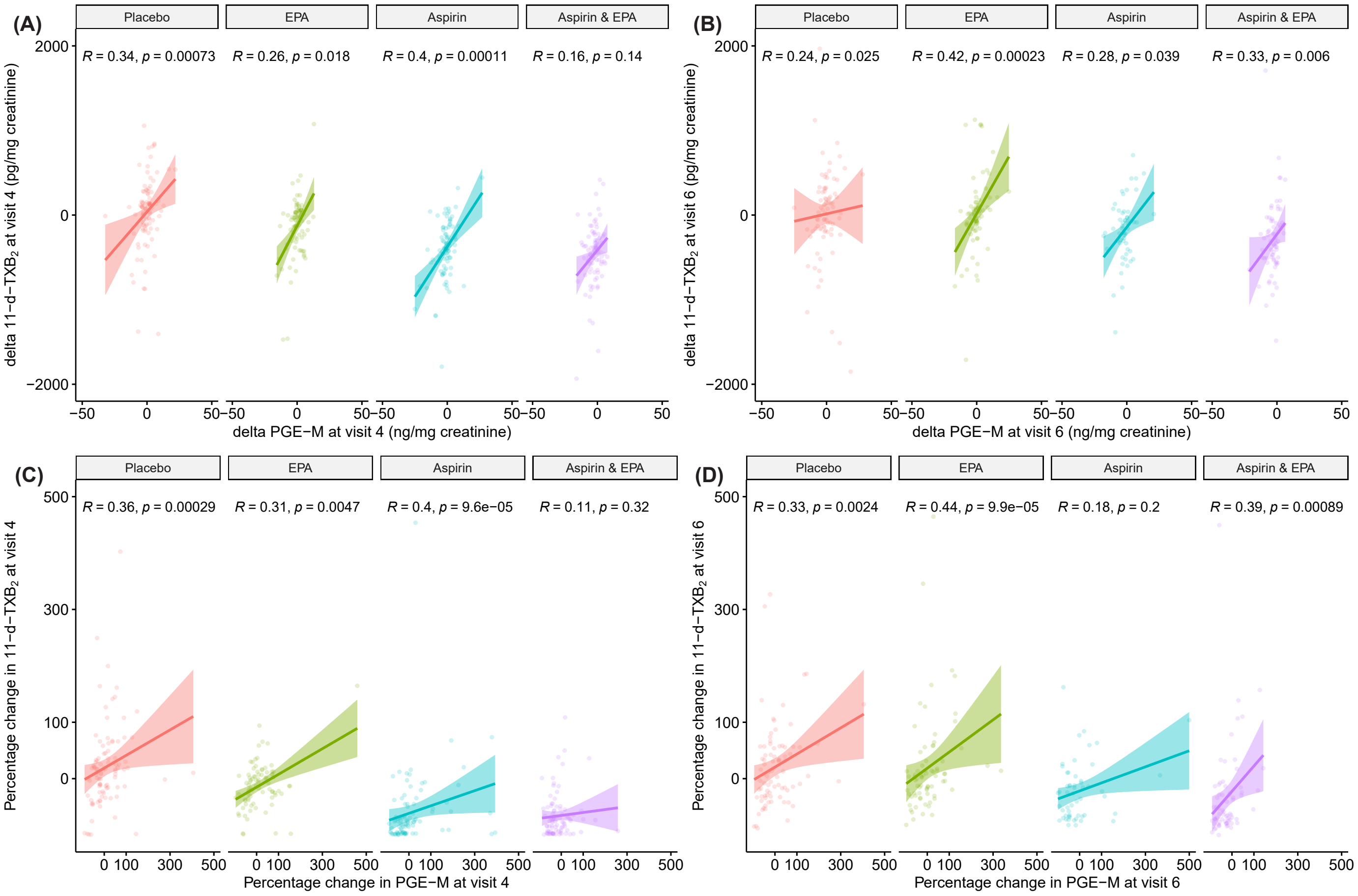

Supplementary Figure 3. Relationship between the change in uPGE-M concentration and respective change in u11-d-TXB_2 concentration compared with the baseline (V1) value in seAFOod trial participants at either V4 (A, C) or V6 (B, D). Data are presented as the absolute change (delta value) in concentration (A, B) or as the % change from baseline (C, D) for the four treatment groups. Spearman's test was used to investigate the relationship between paired urinary biomarker value differences over time.

(A)

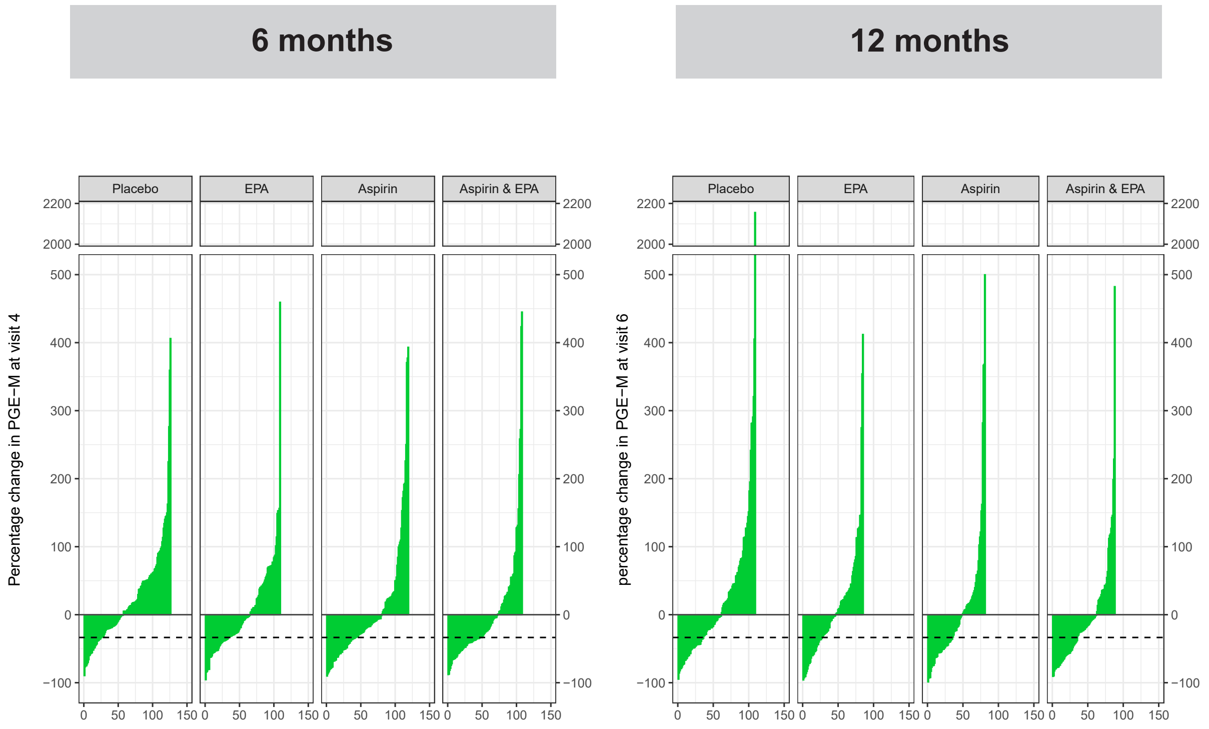

(B)

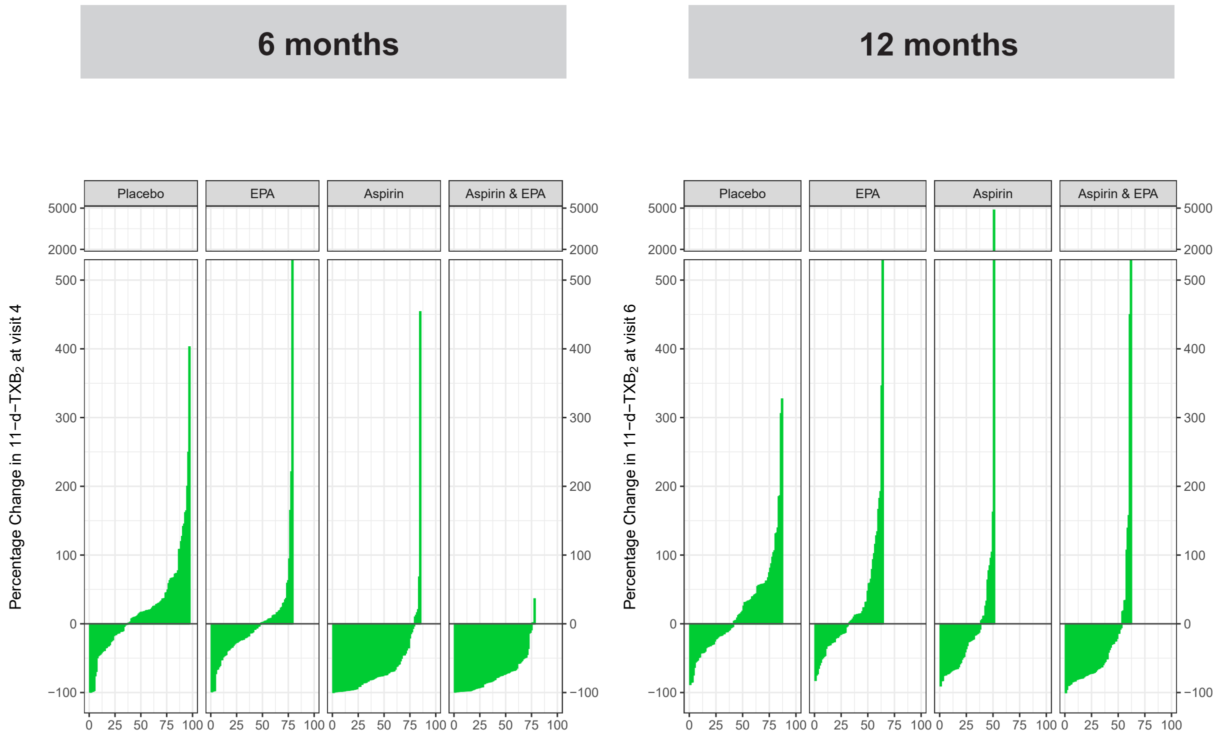

Supplementary Figure 4. The percentage change in uPGE-M level (A) and u11-d-TXB_2 level (B) at 6 months (V4) and 12 months (V6) in seAFOod trial participants according to treatment group, excluding participants reported to be not taking IMP at V4 and/or V6. The X axis denotes the number of participants in each group with paired urinary biomarker data. Individual treatment group summary data and statistical comparisons are presented in **Supplementary Table 3**.

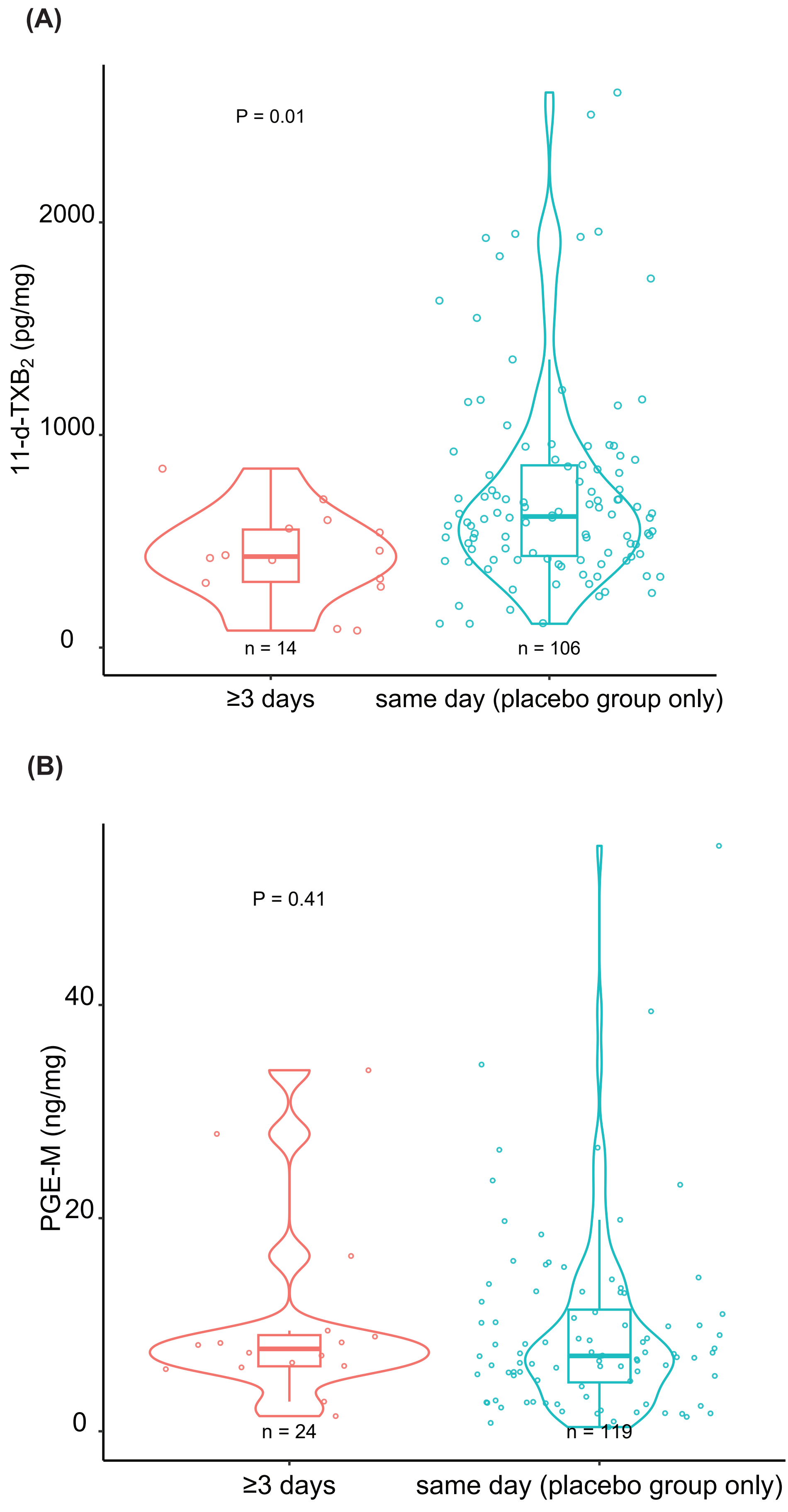

Supplementary Figure 5. Urinary 11-d-TXB_2 (A) and PGE-M (B) levels at V6 comparing participants who provided a urine sample on the same day (after the procedure) as the trial exit colonoscopy (placebo group only) with individuals who provided a urine sample three or more days after the colonoscopy. Data are presented as violin plots with embedded box & whiskers to demonstrate median and IQR values. The statistical significance of the difference between the two groups was tested by the Mann Whitney U test for 11-d-TXB_2 data and the Student's t test for log-transformed PGE-M data.

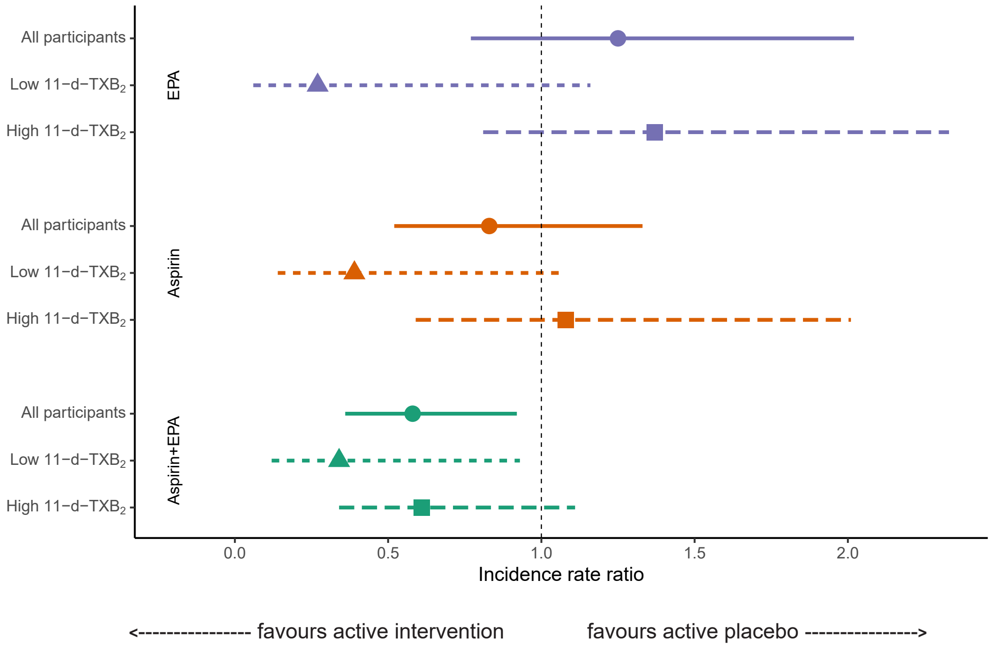

Supplementary Figure 6. The incidence rate ratio estimate and 95% confidence interval for total colorectal polyp number at 12 months in each active treatment group compared with placebo-only users according to the on-treatment u11-d-TXB_2 value. Low u11-d-TXB_2 = quartile 1; High u11-d-TXB_2 = quartile 2, 3 and 4 of V4 (6 months on treatment) values.
